# miR-518f-5p decreases tetraspanin CD9 protein levels and differentially affects non-tumourigenic prostate and prostate cancer cell migration and adhesion

**DOI:** 10.18632/oncotarget.23118

**Published:** 2017-12-07

**Authors:** Danielle R. Bond, Crystal Naudin, Adam P. Carroll, Belinda J. Goldie, Joshua S. Brzozowski, Helen M. Jankowski, Murray J. Cairns, Leonie K. Ashman, Christopher J. Scarlett, Judith Weidenhofer

**Affiliations:** ^1^ School of Biomedical Science and Pharmacy, The University of Newcastle and Hunter Medical Research Institute (HMRI), NSW, Newcastle, Australia; ^2^ Department of Biochemistry and Molecular Biology, Monash University, Clayton, Australia; ^3^ Department of Pediatrics, Emory University, Atlanta, GA, USA; ^4^ School of Environmental and Life Sciences, The University of Newcastle and Hunter Medical Research Institute (HMRI), NSW, Newcastle, Australia; ^5^ Schizophrenia Research Institute, Sydney, NSW, Australia

**Keywords:** miR-518f-5p, prostate cancer, CD9, transcript regulation, metastasis

## Abstract

Tetraspanin CD9 is generally considered to be a metastasis suppressor, with decreased levels associated with progression and metastasis in many advanced stage cancers. Little is known about the cause of CD9 dysregulation in prostate cancer, however there are several miRNA-binding sites in the 3´UTR of the transcript suggesting it could be post-transcriptionally regulated. Using microarrays and luciferase assays in tumourigenic and non-tumourigenic prostate cell lines we identified miR-518f-5p as a regulator of the *CD9 3'UTR* gene expression, and decreased expression of endogenous CD9 in non-tumorigenic prostate RWPE1 and prostate cancer DU145 cells. This resulted in differential functional effects, in which RWPE1 cells showed increased migration and decreased adhesion to extracellular matrix substrates, whereas DU145 cells showed decreased migration and increased adhesion. Moreover, overexpression of miR-518f-5p significantly increased proliferation between 48h and 72h in normal RWPE1 cells, with no effect on tumourigenic DU145 cell proliferation. These results show that tetraspanin CD9 is regulated by miRNAs in prostate cell lines and that due to differential functional effects in non-tumourigenic versus tumourigenic prostate cells, miR-518f-5p may be an effective biomarker and/or therapeutic target for prostate cancer progression.

## INTRODUCTION

Prostate cancer affects 1 in 7 men worldwide [1], with around 5% of all new cases diagnosed with advanced stage metastatic prostate cancer for which the 5-year survival rate is only 30% [2], reflecting the lack of curative therapies for this group. Therefore, novel treatments for prostate cancer that inhibit or slow metastasis are urgently needed. Several members of the tetraspanin family of proteins have been implicated in cancer, particularly metastatic cancers [3] and are potential novel drug target candidates to inhibit prostate cancer progression. In particular, tetraspanin CD9 is commonly referred to as a metastasis suppressor, as reduced CD9 protein levels are associated with advanced stage disease and metastasis [4] and correlate with poor patient prognosis (see review [3]).

To date only two studies have considered the *in vivo* effects of altered CD9 expression in prostate cancer progression showing conflicting results [5, 6]. Overexpression of CD9 in a metastatic prostate cancer cell line increased its invasiveness *in vitro*, but had no effect on tumorigenicity or metastasis in xenograft models [5]. In contrast, CD9 knockout mice crossed onto the TRAMP prostate cancer mouse model led to a significant decrease in spontaneous metastasis to the liver [6], thereby implicating CD9 in prostate cancer progression and metastasis. The discrepancy between these two studies warrants further investigation of CD9 in prostate cancer to clarify if CD9 acts as a metastasis suppressor.

There is little known about regulation of CD9 expression. *CD9* mRNA modifications involving deletions or missense mutations have been found in a small percentage of prostate cancer patients and some prostate cancer cell lines, with CD9 protein not detected in the majority of these cases [4]. Moreover, in Merkel cell carcinoma cell lines *CD9* has mRNA species with differing 5’UTR lengths, with the longer 5’UTR inhibiting ribosome scanning and translation, which may explain the decreased levels of CD9 in Merkel cell carcinoma metastases [7]. DNA methylation in multiple myeloma has been shown to decrease CD9 levels [8], and recently MYCN and HDAC5 were found to decrease *CD9* transcription, leading to invasion and metastasis in neuroblastoma [9]. However, regulation of CD9 in prostate cancer cells remains to be fully elucidated. Expression of miRNAs is also known to be dysregulated in many cancers, including prostate cancer, leading to altered post-transcriptional regulation of their mRNA targets (see review [10]).

Given that CD9 levels and miRNAs are typically altered in prostate cancer, we hypothesized that CD9 may be regulated by miRNAs in prostate cancer, and this may contribute to cancer progression. This study aimed to investigate the influence of miRNA on CD9 levels and determine if miRNAs are responsible for decreased CD9 protein levels in prostate cancers. In accordance with this hypothesis we identified *miR-518f-5p* as a regulator of CD9 and evaluated the functional relevance of this miRNA to prostate cancer progression *in vitro*.

## RESULTS

### CD9 mRNA and protein levels are not correlated in prostate cell lines

**Figure 1:**
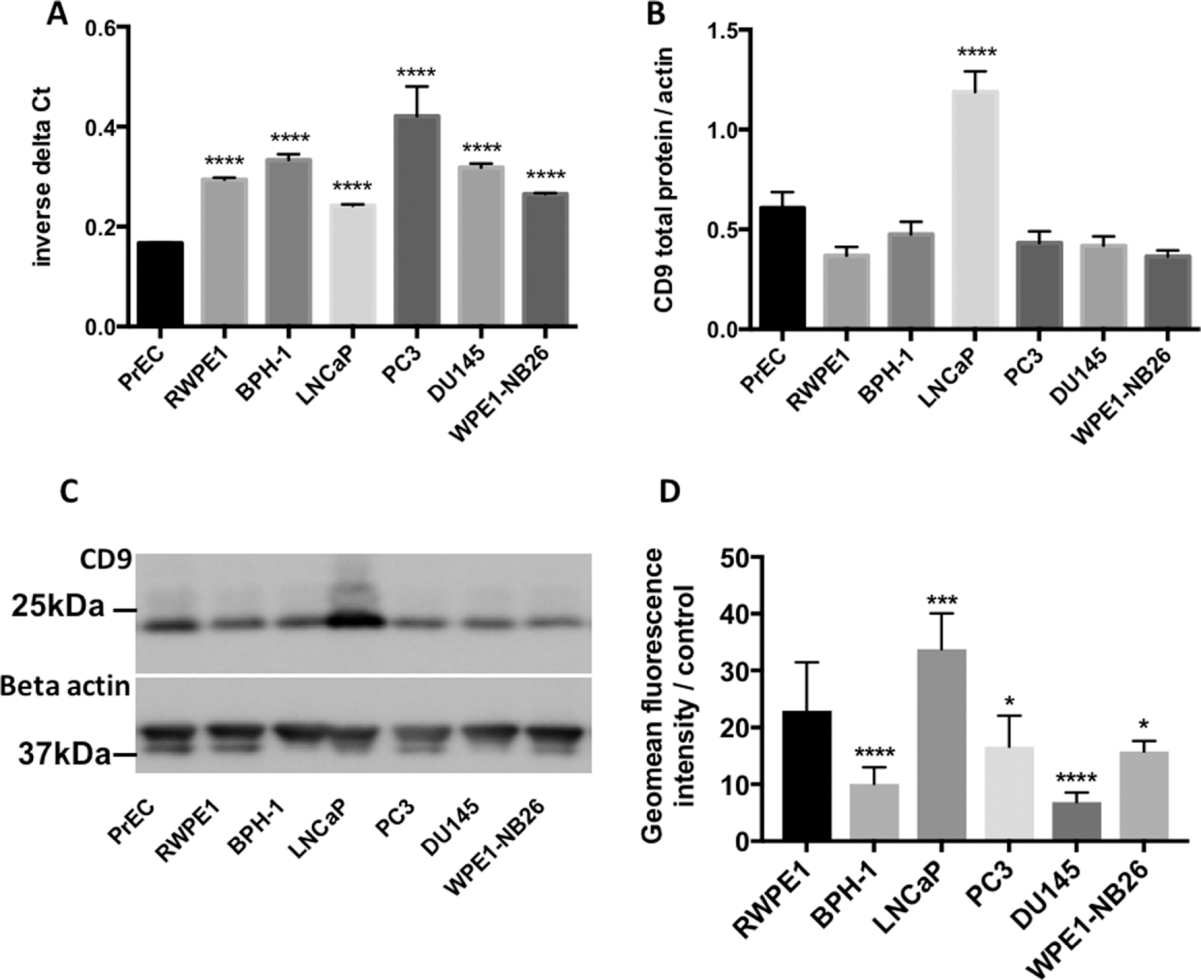
Characterisation of CD9 mRNA and protein levels in prostate cell lines CD9 mRNA (**A**) and total protein levels (**B**) were quite similar across the prostate cell line panel, however LNCaP cells displayed significantly higher levels of CD9 total protein compared to RWPE1 cells. (**C**) Representative western blot showing CD9 total protein levels across prostate cell lines. (**D**) Flow cytometry analysis revealed that CD9 cell surface levels were significantly increased in LNCaP cells and significantly decreased in BPH-1, DU145, PC3 and WPE1-NB26 cells compared to RWPE1 cells. All experiments were conducted with *n* = 3; y-axes of graphs are shown as arbitrary units, *p* = 0.05^*^, *p* = 0.001^***^, *p* < 0.0001^****^.

**Figure 2:**
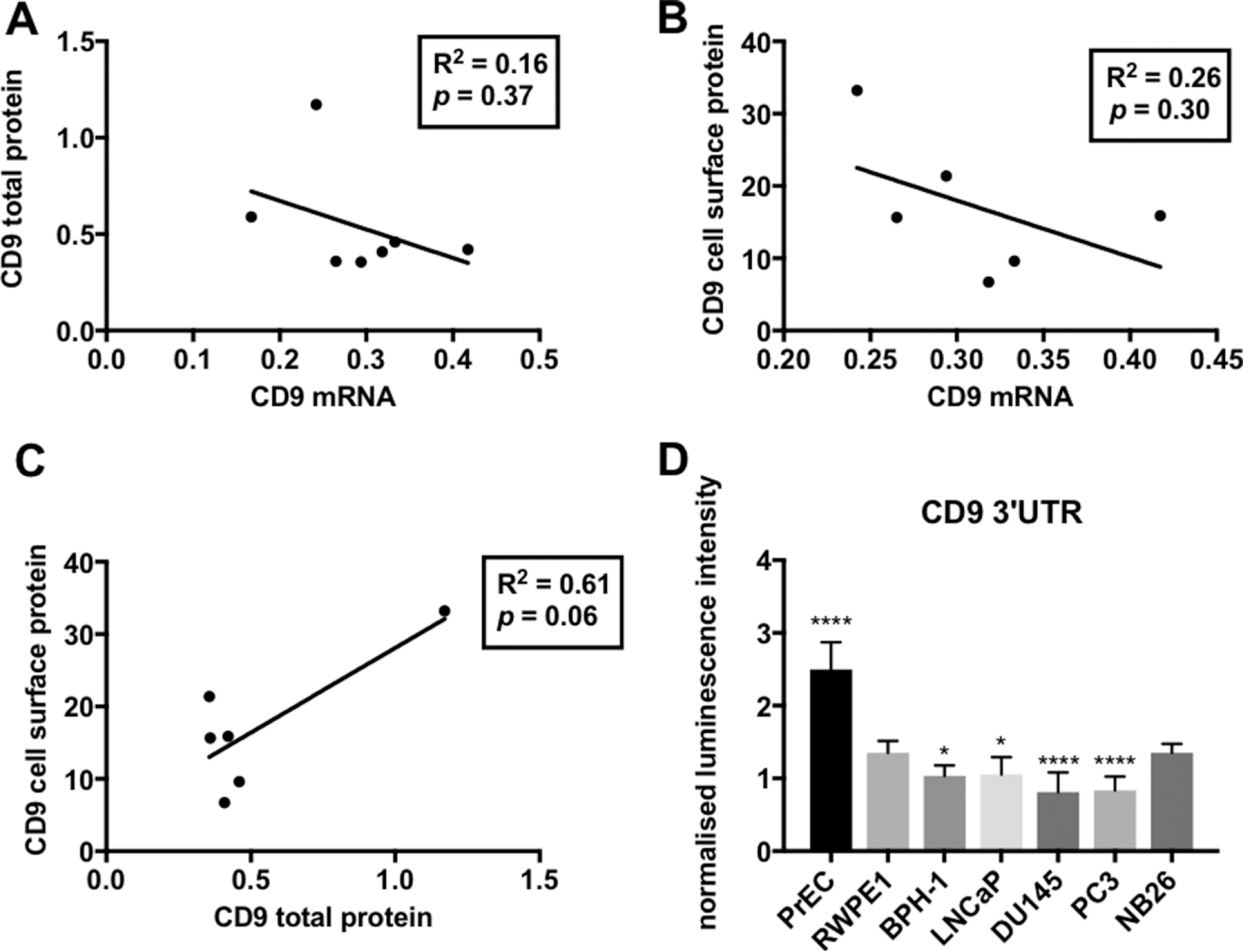
Linear regression analysis of CD9 levels in prostate cell lines (**A**) An inverse trend was found between the geomean of CD9 mRNA levels versus the geomean of CD9 total protein levels (from Figure 1). (**B**) An inverse trend between the geomean of CD9 mRNA levels compared to geomean of CD9 cell surface levels was observed. (**C**) A positive trend was seen between the geomean of CD9 total protein levels versus geomean of CD9 cell surface levels (*p* = 0.06). For all combinations (A, B and C), a linear regression analysis was performed with 95% CI and *p* < 0.05. All results are shown as arbitrary units. (**D**) Prostate cancer cell lines (PC3 and DU145) naturally display repressed CD9 3’UTR activity compared to non-tumorigenic prostate cells as assessed using a CD9 3’UTR dual luciferase reporter assay. Renilla luminescence intensity was normalized to a firefly luciferase vector (transfection control) and the 3’UTR empty vector (positive control; *n* = 3, y-axis shown as arbitrary units; *p* = 0.01^*^, *p* < 0.0001^****^.

The level of CD9 mRNA and protein was determined in a panel of non-tumorigenic (PrEC, RWPE1 and BPH-1) and tumorigenic (LNCaP, PC3, DU145 and WPE1-NB26) prostate cell lines. Whilst there was little variation in *CD9* mRNA levels, more aggressive prostate cancer cell lines (DU145 and PC3) displayed the highest levels of *CD9* mRNA (Figure 1A). In contrast, the aggressive prostate cancer cells had the lowest levels of CD9 cell surface protein (Figure 1D) and total CD9 protein levels compared to non-tumorigenic RWPE1 cells (Figure 1B and 1C). Moreover, the lowly tumorigenic LNCaP cells had the highest levels of CD9 cell surface and total protein (Figure 1B–1D). *CD9* mRNA and CD9 total protein or cell surface protein levels showed an inverse correlation that did not reach significance (Figure 2A and 2B), and CD9 total protein and cell surface protein levels showed a trend towards a positive correlation (Figure 2C). However, when the outlier (LNCaP) is removed, there is no trend towards a correlation between CD9 protein and cell surface levels (Supplementary Figure 1).

### Post-transcriptional regulation of CD9 varies across prostate cell lines

The contribution of post-transcriptional regulation of *CD9* levels at the 3’UTR via endogenous factors was shown through transfection of renilla luciferase gene fused with the *CD9* 3’UTR into the panel of prostate cell lines. The aggressive DU145 and PC3 prostate cancer cells displayed reduced luciferase activity with the *CD9* 3’UTR compared to RWPE1 control (Figure 2D), which is indicative of endogenous regulatory factors acting within these cells to repress CD9 levels. In comparison, the other prostate cell lines showed minimal (BPH-1 and LNCaP) or no (WPE1-NB26) 3’UTR repression, or enhanced luciferase activity (PrEC) due to the presence of the *CD9* 3’UTR compared to RWPE1 control cells (Figure 2D).

### miRNAs predicted to regulate the *CD9* 3’UTR show differential expression in prostate cancer cell lines

**Figure 3:**
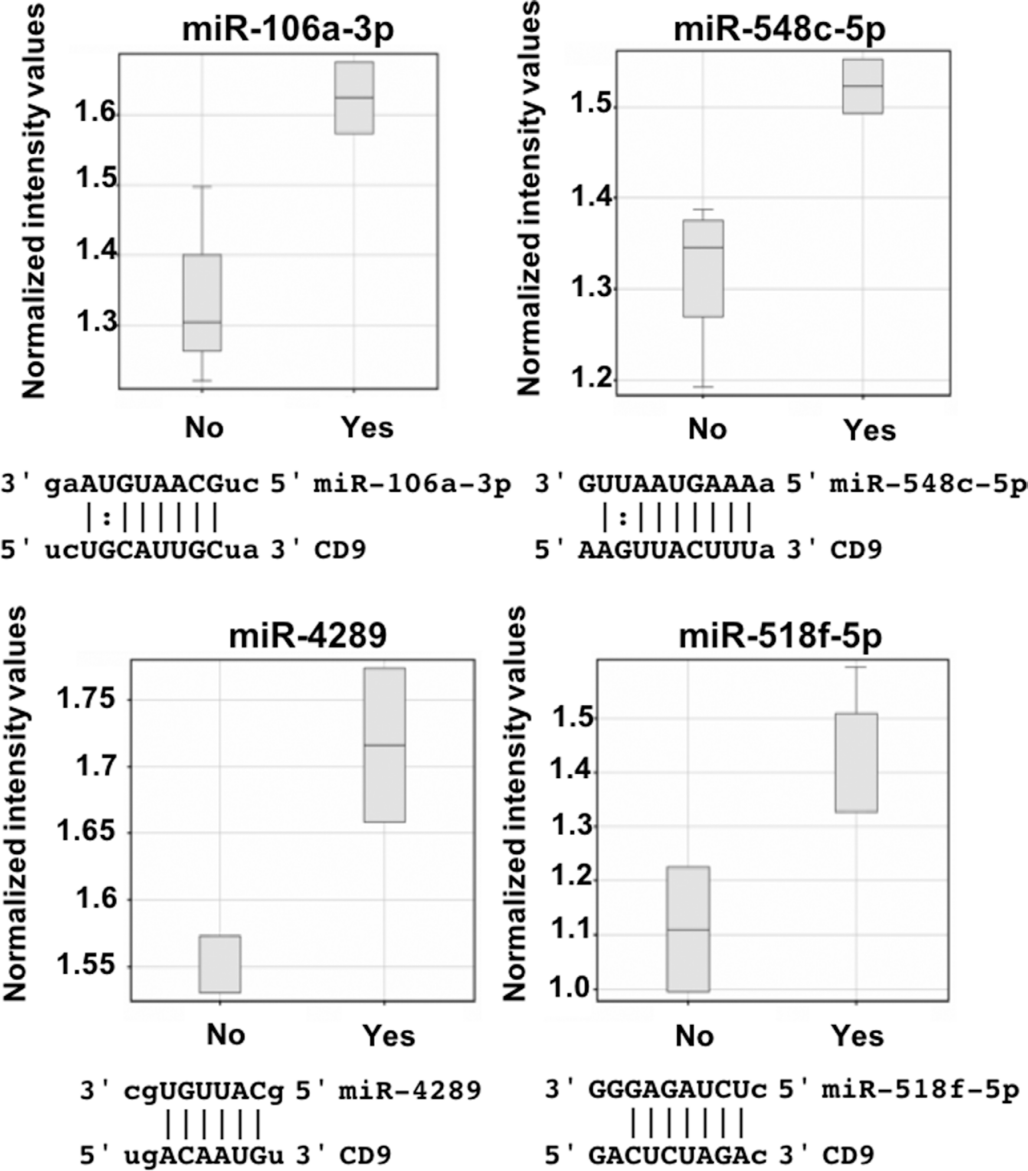
miR-106a-3p, miR-548c-5p, miR-4289 and miR-518f-5p are upregulated in prostate cells showing changes to *CD9* 3’UTR activity GeneSpring software was used to analyse miRNA expression patterns, with miR-106a-3p, miR-548c-5p, miR-4289 and miR-518f-5p showing increased levels in prostate cell lines that have changes to CD9 3’UTR activity, and are predicted to bind to the CD9 3’UTR. All results are shown as normalized intensity values (arbitrary units); yes represents having displayed changes to CD9 3’UTR activity compared to controls and no represents no change compared to controls. CD9/miR sequence alignments from microrna.org are shown below each miR box plot.

**Figure 4:**
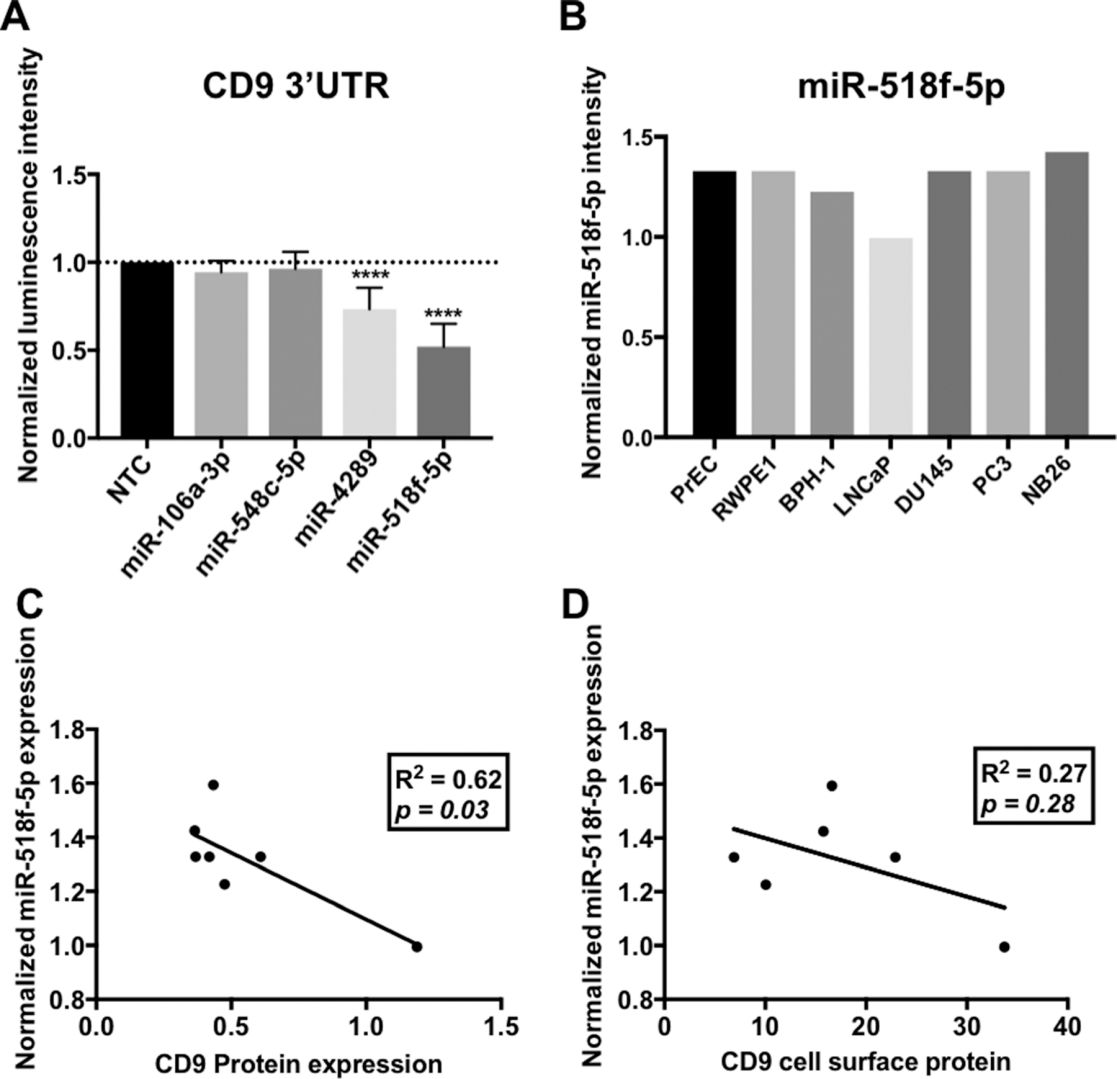
miRNAs predicted to regulate *CD9* bind to the CD9 3’UTR *in vitro* (**A**) Transfection of miR-106a-3p and miR-548c-5p mimics led to no change in CD9 3’UTR activity, however miR-4289 and miR-518f-5p resulted in a significant decrease in CD9 3’UTR activity; *n* = 3, results expressed in arbitrary units; *p* < 0.0001^****^. Results are shown as luminescence intensity normalized to firefly luminescence (transfection control) and expressed as a fold change relative to a miRNA negative control (NTC). (**B**) miR-518f-5p shows differential expression in prostate cell lines as assessed by miRNA microarrays. Results are shown as normalized intensity values, *n* = 1. (**C** and **D**). Linear regression analysis of miR-518f-5p levels and CD9 total protein (C) and CD9 cell surface levels (D). A significant inverse correlation was found between miR-518f-5p and CD9 total protein levels, however a trend towards a negative correlation was only observed for miR-518f-5p and CD9 cell surface protein levels.

To identify which endogenous miRNA contribute to the *CD9* 3’UTR regulation identified with the luciferase assay, miRNA profiles were generated using Affymetrix miRNA microarrays. Using GeneSpring GX software, miRNA that showed differential expression between cell lines grouped by the amount of endogenous targeting of the *CD9* 3’UTR that was observed with the luciferase assay (Figure 2D) were identified. The miRNA were prioritised based on being predicted to bind to, and regulate the *CD9* 3’UTR as identified by microRNA.org [11]. Increased expression of miR-106a-3p, miR-548c-5p and miR-4289 was found in prostate cell lines that displayed low luciferase levels due to *CD9* 3’UTR repression (PC3 and DU145; Figure 3). Alternatively, grouping the cell lines as either showing decreased luciferase (PC3 and DU145), or enhanced luciferase activity (PrEC, RWPE1 and WPE1-NB26) compared to no change (BPH-1 and LNCaP) identified miR-518f-5p as having an expression profile (Figure 4B) that matched the luciferase output (Figure 2D), and was also predicted to bind to the *CD9* 3’UTR (Figure 3). Additionally, when compared to CD9 protein levels, a significant inverse correlation was found between miR-518f-5p and total protein levels (Figure 4C), and an inverse trend was seen between miR-518f-5p and CD9 cell surface protein levels (Figure 4D).

miRNAs identified as having the potential to regulate *CD9* at the 3’UTR (miR-106a-3p, miR-548c-5p, miR-4289 and miR-518f-5p) were co-transfected with the *CD9* 3’UTR luciferase construct in HEK293FT cells, to determine if they modified luminescence intensity. miR-106a-3p and miR-548c-5p had no significant effect on the *CD9* 3’UTR, whilst miR-4289 and miR-518f-5p significantly decreased luciferase activity resulting from the *CD9* 3’UTR constructs by 25% and 40% respectively, compared to non-targeting control (NTC) miRNA mimics (Figure 4A).

### miR-518f-5p decreases CD9 protein levels in prostate cell lines

**Figure 5:**
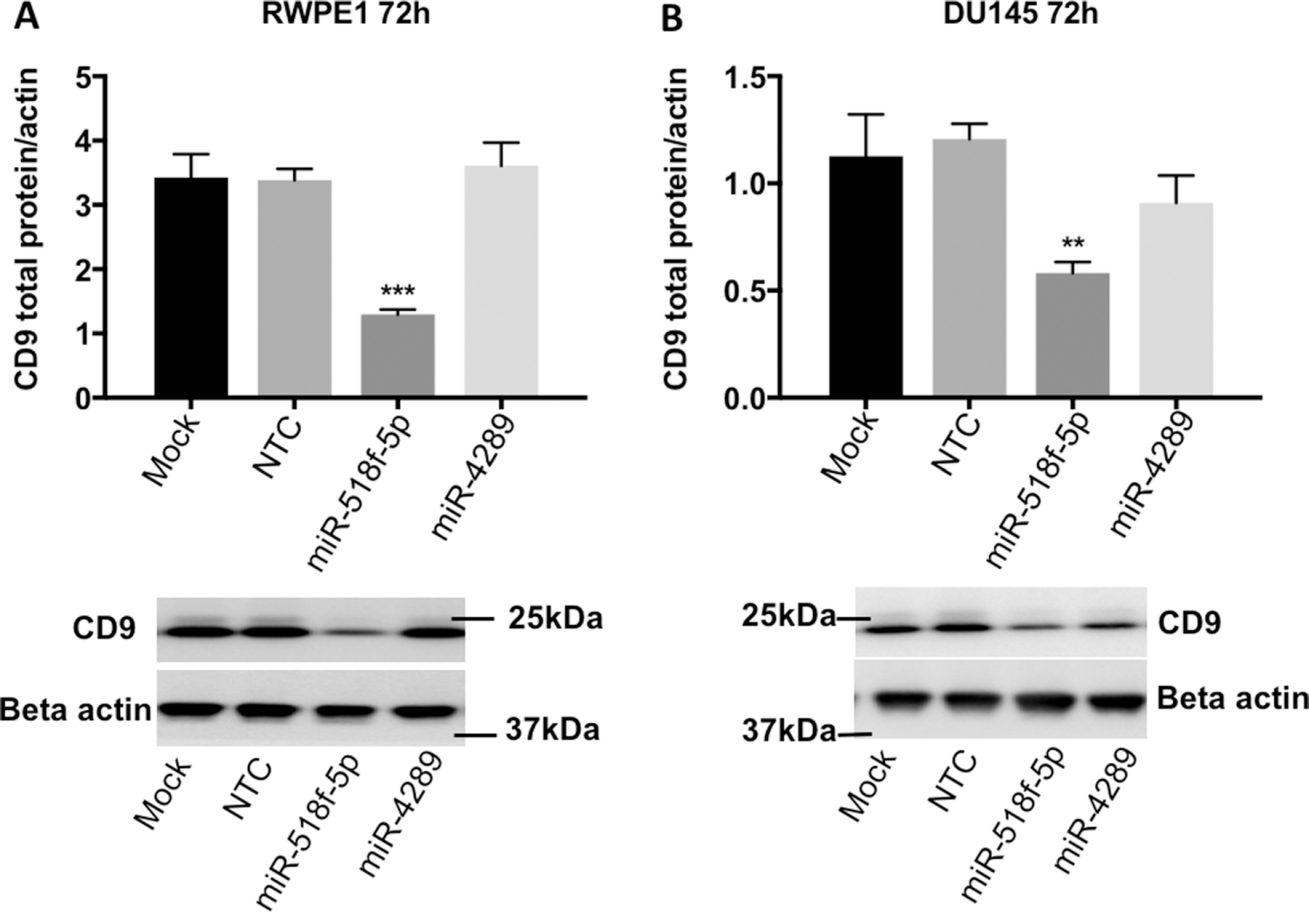
miR-518f-5p decreases CD9 protein levels in RWPE1 and DU145 prostate cells Transfection with miR-518f-5p significantly reduced CD9 protein levels in RWPE1 cells 72h post-transfection (*p* = 0.0004^***^), whereas miR-4289 had no effect on CD9 protein levels (*p* = 0.6018), compared to NTC (**A**). Transfection with miR-518f-5p significantly reduced CD9 protein levels in DU145 prostate cancer cells at 72h post-transfection (*p* = 0.002^**^) compared to NTC, whereas miR-4289 led to a slight reduction in CD9 protein levels that did not reach significance (*p* = 0.1102) (**B**). Results are shown as CD9 total protein normalized to beta actin protein levels (arbitrary units), which served as the loading control; *n* = 3. Representative western blots are shown below each graph depicting quantitated protein levels.

As miR-518f-5p and miR-4289 showed the greatest capacity to regulate the CD9 3’UTR, their ability to alter CD9 levels in prostate cancer cells and the resultant change in cell function was investigated. Transfection of miR-4289 mimic, had no significant effect on CD9 total protein levels in RWPE1 cells at 72h (106%, *p* = 0.6018), or DU145 prostate cancer cells at 72h (75.8%, *p* = 0.1102) and so was not assessed further (Figure 5). Whereas transfection of miR-518f-5p mimic into RWPE1 and DU145 prostate cell lines resulted in a significant decrease in CD9 total protein levels in RWPE1 cells at 72h (61.7%, *p* = 0.0004) and in DU145 cells at 72h (51.95%, *p* = 0.002) compared to cells transfected with NTC (Figure 5). Assessing CD9 cell surface expression after transfection of the miR-518f-5p mimic showed a 62.3% decrease in RWPE1 cells (*p* = 0.0001) and a 12.1% decrease in DU145 cells (*p* = 0.0196) compared to NTC at 72h (data not shown).

### miR-518f-5p modulates prostate cell migration, adhesion and proliferation

**Figure 6:**
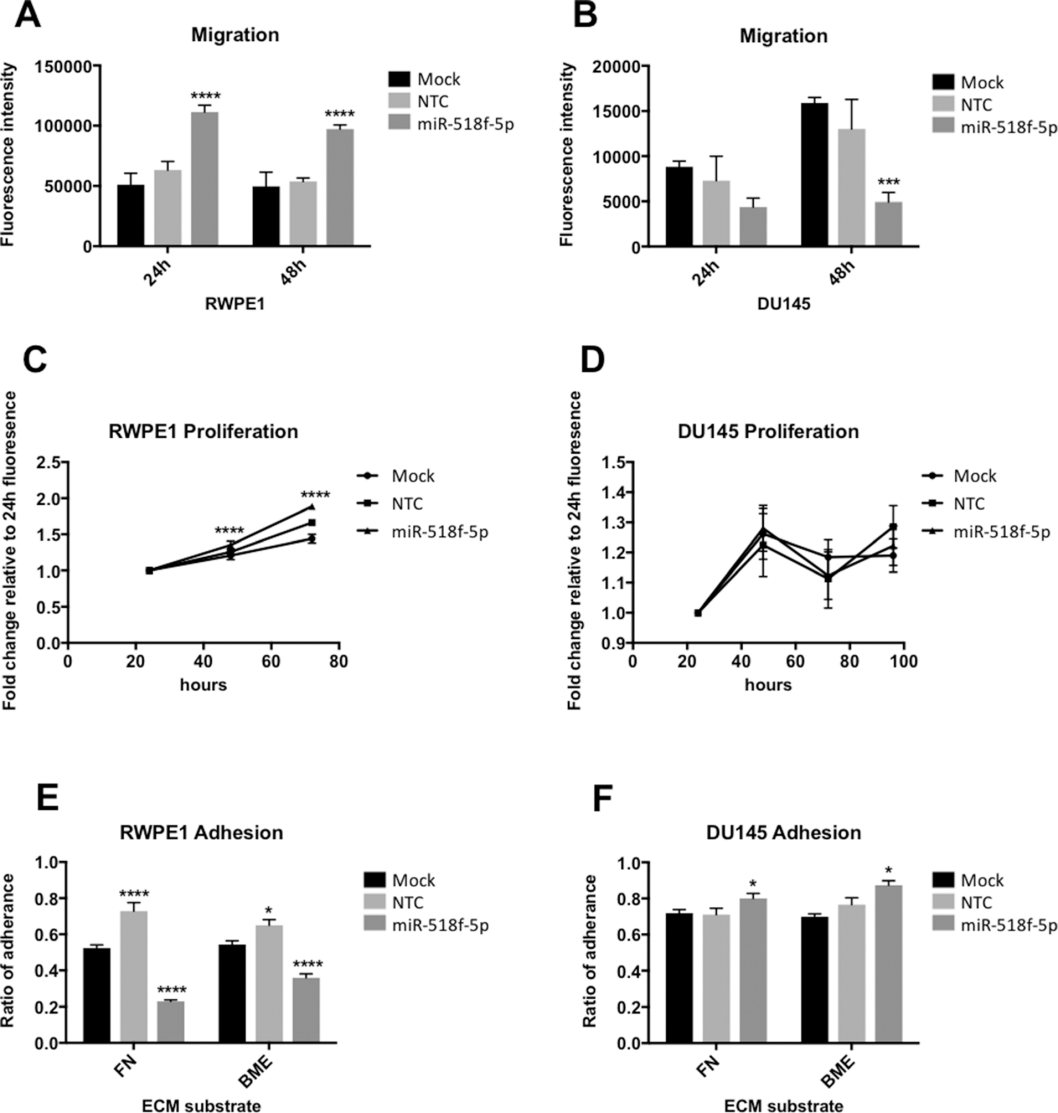
miR-518f-5p differentially affects migration and adhesion of prostate cell lines (**A**) Transfection of miR-518f-5p Significantly increased RWPE1 migration at 24h (*p* = 0.0001^****^) and 48h (*p* = 0.0001^****^) compared to NTC. (**B**) miR-518f-5p significantly decreased DU145 prostate cancer cell migration at 48h compared to NTC (*p* = 0.0004^***^). Results are shown as fluorescence intensity of migratory cells normalized to fluorescence intensity of RWPE1 or DU145 cells prior to commencement of the assay. (**C** and **D)**. miR-518f-5p significantly increased RWPE1 cell proliferation at 48h (*p* = 0.0001^****^) and 72h (*p* = 0.0001^****^), but had no substantial effect on DU145 cell proliferation compared to NTC. Results are shown as fold change fluorescence intensity relative to 24h fluorescence. (**E**) miR-518f-5p significantly decreased RWPE1 cell adhesion to fibronectin (FN) (*p* = 0.0001^****^) and basement membrane extract (BME) (*p* = 0.0001^****^) compared to NTC. (**F**) miR-518f-5p significantly increased DU145 cell adhesion to FN (*p* = 0.0469^*^) and BME (*p* = 0.0140^*^). Results are expressed as ratio of adherence which is fluorescence intensity of adherent cells/fluorescence intensity of total cells. All experiments consisted of mock cells (cells with transfection reagent only) and NTC cells (cells transfected with a scrambled non-targeting miRNA mimic negative control); *n* = 3 and all y-axes are shown as arbitrary units. Graphs were analysed using ANOVA with Dunnett's multiple testing.

To determine whether miR-518f-5p is involved in cancer-related processes involving CD9, miR-518f-5p mimic was transiently transfected into non-tumorigenic RWPE1 and metastatic DU145 prostate cancer cells, and their proliferative, migratory and adhesive capacity assessed post-transfection. Reduced CD9 levels over the course of the functional assays was confirmed using western blotting at the experimental end point (data not shown). Transfection of miR-518f-5p significantly increased migration of RWPE1 cells at both 24h and 48h (*p* = 0.0001) (Figure 6A), but significantly decreased migration in DU145 prostate cancer cells at 48h (*p* = 0.0004) (Figure 6B). There was a significant increase in RWPE1 cell proliferation following transfection of miR-518f-5p compared to NTC at 48h (*p* = 0.0001) and 72h (*p* = 0.0001) (Figure 6C), however there was no substantial effect on DU145 cell proliferation between 24h and 96 h (*p* = 0.99 (24 h); *p* = 0.15 (48 h); *p* = 0.92 (72 h); *p* = 0.09 (96 h)) (Figure 6D). Furthermore, a differential effect on cell adhesion to fibronectin (FN) and basement membrane extract (BME) at 1h was observed, with transfection of miR-518f-5p leading to a significant decrease in RWPE1 cell adhesion to both substrates (*p* = 0.0001) (Figure 6E) compared to a significant increase in DU145 cell adhesion to FN (*p* = 0.0469) and BME (*p* = 0.014) compared to NTC cells (Figure 6F).

## DISCUSSION

Tetraspanin CD9 plays a crucial role in cancer progression and metastasis and its protein expression is typically down-regulated in advanced stage and metastatic prostate cancer. Given the potential clinical significance of CD9 in the prevention of transition to a more invasive tumor phenotype, we believe that modulation of CD9 expression may serve as a new therapeutic strategy whereby expression could be supported prior to tumor progression or reactivated in advanced disease stages. However, this requires an understanding of the mechanism/s by which CD9 expression and function is regulated in non-tumourigenic prostate and prostate cancer cells, which we have partially addressed with this study.

In contrast to expectation, there was minimal change in CD9 total and cell surface protein levels across the panel of tumourigenic and non-tumourigenic prostate cells and no correlation between CD9 mRNA and protein levels. However, prostate cell lines with high *CD9* mRNA typically displayed low levels of CD9 protein, suggesting that CD9 may be post-transcriptionally regulated by miRNAs. Furthermore, the level of luciferase activity as regulated by the *CD9* 3’UTR, showed that endogenous factors worked to different extents within this panel of cell lines to modulate expression via the *CD9* 3’UTR. This is an important consideration in terms of tumor function, as in addition to loss of CD9 expression, factors that act at the 3’UTR have great capacity to affect many proteins leading to widespread functional differences. Using microarrays to assess global differences in miRNA expression in the cell lines, miR-518f-5p was identified as a potential modulator of CD9 expression, which was then confirmed via transfection of miR-518f-5p mimic.

In contrast to what is typically observed in prostate cancer, LNCaP cells, which are derived from a lymph node metastasis of prostate cancer, had high levels of *CD9* mRNA, CD9 total and cell surface protein. Up-regulation of CD9, especially on the cell surface, has been shown to serve an important role in communication between tumor cells and the microenvironment, allowing trans-endothelial migration and hence metastasis, which opposes the notion that CD9 functions as a generic metastasis suppressor [12]. Therefore, LNCaP cells may have increased expression of CD9 in preparation to traverse into the circulation from the lymphatic system.

Cell surface levels of CD9 across the panel of prostate cell lines was as expected for a metastasis suppressor. However, this was not the case for *CD9* mRNA and total protein levels, with non-tumorigenic cells having similar levels to prostate cancer cell lines. Whilst decreased total CD9 levels are frequently observed in most solid malignancies, decreased cell surface CD9 protein levels may be more relevant, as this population of CD9 is involved in forming tetraspanin enriched microdomains (TEMs), which are involved in cell signalling [3, 13, 14]. Decreased CD9 on the cell surface would disrupt the stoichiometry of TEMs and thereby signalling from the membrane into the cell. Interestingly, cell surface CD9 expression following transfection of miR-518f-5p mimic showed a stark difference in abundance of CD9 between the two cell lines, with the non-tumourigenic RWPE1 showing a 62% reduction whereas the tumourigenic DU145 showed only a 12% reduction. It is therefore likely this differential effect would have contributed to the functional effects on proliferation, migration and adhesion observed between the cell lines despite both cell lines showing greater than 50% reduction in total protein. Whilst not assessed in the study by Copeland *et al.* [6], it is also possible that systemic knockout of CD9 in the TRAMP mouse model led to altered activity of miR-518f-5p against its other endogenous targets, and contributed to the significant reduction in metastasis to the liver [6].

Effects on adhesion and migration were not unexpected in the context of CD9, as a previous study showed effects on metastasis *in vivo* [6], however alterations to proliferation have not been reported in normal prostate or prostate cancers [6]. Importantly in the context of novel therapeutics development, it is vital to have selectivity between effects in non-tumourigenic and tumorigenic cells, which we have shown to occur with addition of miR-518f-5p mimic.

CD9 is reported to be important for adhesion to the ECM [14–16]. In this study, significant changes in adhesion were observed in RWPE1 and DU145 cells. Following transfection of the miR-518f-5p mimic, RWPE1 prostate cells showed a significant decrease in initial adhesion to FN and BME, whereas DU145 cells showed a significant increase in adhesion to FN and BME. CD9 has been shown to bind to FN [17], therefore the decrease in CD9 protein levels elicited by transfection of miR-518f-5p may disrupt this interaction, resulting in reduced RWPE1 cell adhesion to FN. This has also been observed in RWPE1 cells with stable knockdown of CD9 (Bond *et al.,* unpublished). Moreover, BME is used to recapitulate the ECM *in vitro*, with decreased levels of CD9 resulting in decreased cell adhesion to BME, likely through loss of CD9 signalling through integrins and other TEM proteins, which modulate cell adhesion [3]. It is unclear why miR-518f-5p specifically decreased RWPE1 cell adhesion whilst increasing DU145 cell adhesion to ECM substrates. However, we postulate this is likely due to the already reduced levels of CD9 in DU145 cells, minimal difference in cell surface CD9 abundance compared to total protein levels and the availability of other miR-518f-5p mRNA targets, as well as differences in cell adhesion pathways between these two cell lines.

Transfection of miR-518f-5p also led to a differential change in cell migration, where it enhanced migration of RWPE1 cells, but inhibited migration of DU145 prostate cancer cells. The differential effect of miR-518f-5p on migration of non-tumorigenic prostate cells compared to the prostate cancer cell line is particularly interesting. This is also likely to be influenced by differences in the expression of miR-518f-5p target mRNAs including CD9, as well as the expression of other miRNAs that can work co-operatively with, or against the actions of miR-518f-5p. Moreover, CD9 may be involved in different pathways associated with migration in non-tumorigenic prostate cells compared to prostate cancer cells. There have been no studies investigating the function of miR-518f-5p, and its mRNA targets are currently unknown. Publically available target prediction databases suggest genes involved in cancer related pathways, such as Wnt signalling, integrin signalling and angiogenesis, as miR-518f-5p targets (miRpath [18]). Interestingly, many proteins from these pathways are also CD9 or other tetraspanin protein partners and are all involved in cancer progression. Therefore, the results from this study suggest that miR-518f-5p may play an important role in prostate cancer progression via modulation of migration and adhesion through regulation of CD9 expression and potentially a range of other cancer related proteins.

In this study, CD9 mRNA and protein levels were found to be similar in non-tumorigenic and prostate cancer cell lines. However, most prostate cancer cells had decreased CD9 cell surface levels compared to non-tumorigenic prostate cells. Bioinformatics, miRNA profiling and a *CD9* 3’UTR luciferase reporter assay inferred that miRNAs regulated CD9 expression. miR-518f-5p was predicted to regulate CD9, was found to bind to the *CD9* 3’UTR *in vitro* and transfection of miR-518f-5p significantly reduced CD9 protein levels. Moreover, transfection of miR-518f-5p led to a differential effect on migration and adhesion. In the RWPE1 non-tumorigenic prostate cell line migration was promoted and adhesion decreased, whilst in the DU145 tumourigenic prostate cancer cell line migration was reduced and adhesion increased, therefore, miR-518f-5p could potentially play an important role in prostate cancer progression and be a novel therapeutic target to inhibit cancer metastasis.

## MATERIALS AND METHODS

### Cell lines and maintenance

Primary human prostate epithelial PrEC cells (CC-2555, Lonza, Basel, Switzerland) were cultured in PrEBM with supplements (Lonza) as per manufacturer's recommendations. RWPE1 (CRL-11609), WPE1-NB26 (CRL-2852), PC3 (CRL-1435), LNCaP (CRL-1740) and DU145 (HTB-81) were purchased from and authenticated by ATCC (ATCC, Virginia, USA). RWPE1 and WPE1-NB26 cells were cultured in complete Keratinocyte Serum-Free Media (K-SFM) (Invitrogen, California, USA). BPH-1, a kind gift from Prof Gail Risbridger (Monash University, VIC, AUS), HEK293FT, a kind gift from Prof John Aitken (University of Newcastle, NSW, AUS), PC3, DU145 and LNCaP cells were all cultured in RPMI-1640 (GE Healthcare, Utah, USA) supplemented with 10% FBS (Sigma-Aldrich, Missouri, USA) and 2mM L-glutamine (GE Healthcare). All cells were maintained at 37°C with 5% CO_2._ All cell lines except BPH-1 were used within 4 years of purchase from ATCC, BPH-1 cells were authenticated using the GenePrint 10 System (Promega, Wisonsin, USA) as per manufacturer's instructions and DNA fragments detected by the Australian Genome Research Facility (AGRF) (VIC, AUS).

### CD9 3’UTR dual luciferase reporter assay

Prostate cells were co-transfected with *CD9* 3’UTR or empty 3’UTR renilla reporter vector (100ng) (SwitchGear Genomics, California, USA) and 10ng pmiR-Report firefly luciferase transfection control vector (Promega) using Lipofectamine LTX (Invitrogen, California, USA). Luciferase activity was assessed 24h post-transfection using the dual luciferase assay kit (Promega) and a Biotek Synergy 2 plate reader. Results are expressed as luminescence intensity normalized to firefly transfection control vector and then empty 3’UTR vector. For assessment of specific miRNA, *CD9* 3’UTR luciferase reporter vector and 50mM miRNA mimics (Bioneer Pacific, VIC, AUS) were co-transfected into HEK293FT cells.

### Transient reverse transfection of miRNA mimics

miRNA mimics (100 nM) were reverse transfected using Lipofectamine RNAiMAX (Invitrogen) into RWPE1 and DU145 cells and protein isolated at 48h and 72h post-transfection as described below.

### Total RNA extraction and miRNA expression profiling

RNA was extracted with TRIzol reagent (Invitrogen) according to manufacturer's recommendations with precipitation at – 20°C overnight. RNA quantity and integrity was assessed as described previously [19]. Global miRNA expression was analysed by labelling 1μg of RNA with the FlashTag Biotin HSR RNA labelling kit (Genisphere, Pennsylvania, USA), hybridizing to Genechip miRNA 2.0 microarrays (Affymetrix, California, USA) and scanned with Affymetrix GeneChip Scanner 3000 7G. Signals were normalized across all microarray chips using Robust Multi-array Analysis (RMA) (GeneSpring GX software). Data was grouped based on each cell line's level of tetraspanin *CD9* 3’UTR targeting as determined by luciferase assays to identify miRNA likely to target the *CD9* 3’UTR.

### cDNA synthesis and qPCR

Total RNA was reverse transcribed into cDNA using superscript II reverse transcriptase (Invitrogen) in accordance with the manufacturer's recommendations. qPCR was conducted with the ABI7500 (Applied Biosystems, California, USA) using 1x SYBR master mix (SensiMix SYBR kit (Bioline, London, UK)), 300 nM forward and reverse primer mix and 0.75ng of cDNA). Cycling consisted of 50°C for 2min, 95°C for 10min, then 40 cycles of 95°C for 15s, 60°C for 1min. To calculate the delta Ct, the average of the geomean of *HMBS*, *GusB* and *RPS18* was subtracted from the tetraspanin *CD9* Ct values and the results expressed as inverse delta Ct.

### Protein extraction and quantitation

Cellular proteins were solubilised in 1% NP40 lysis buffer (50mM Tris-HCl, 150mM NaCl, 1% NP40 pH8.0, with 1X cOmplete EDTA free protease inhibitor cocktail (Roche, Mannheim, Germany)). Insoluble proteins removed by centrifugation at 15000 rcf for 30min at 4°C and total protein concentration determined with the microBCA kit (Pierce, Rockford, IL, USA).

### SDS-PAGE and western blotting

Protein samples (20μg) were electrophoresed on 4-12% Novex Tris-Glycine pre-cast gels (Life Technologies, California, USA) using non-reducing conditions in 1x MOPS running buffer (Astral Scientific, NSW, AUS) and transferred to Hybond™-C Extra Nitrocellulose membrane (GE Healthcare). Membranes were incubated overnight in TTBS (Tris-buffered saline with 0.1%Tween-20) at 4°C, blocked for 30min in 5% skim milk powder in TTBS. Anti-CD9 primary antibody (clone1AA2 4μg/mL; made in-house, Ashman Laboratory, AUS) or anti-β-actin (1:20000; AC-15; Sigma-Aldrich) were detected with HRPD-conjugated goat anti-mouse secondary antibody (1:5000, Bio-Rad (72-1011), California, USA) and enhanced chemiluminescence (25mL TBS, 2.5mM Luminal, 0.4mM PCA and 2.6mM hydrogen peroxide) with the Fujifilm LAS4000 (Fujifilm, Tokyo, Japan).

### Flow cytometry

Cell surface CD9 expression was assessed at 90% confluency with the CD9 antibody at 8μg/mL and FITC-conjugated goat anti-mouse IgG secondary antibody (1:50; Southern Biotech, Alabama, USA) and analysed using the FACScalibur (BD Biosciences, California, USA). Results are shown as geomean of FITC fluorescence minus geomean of isotype matched control IB5 antibody.

### Cell proliferation assay

Cell proliferation was assessed using the resazurin assay as described previously [20]. Rate of cell proliferation was determined using number of viable cells at 24h, 48h, 72h and 96h, with results presented as fold change of fluorescence intensity relative to 24h fluorescence intensity (arbitrary units).

### Cell migration assay

Migration assays were performed using 6.5mm, 8μm pore size 24-well Transwells™ (Corning, NY, USA). Cells (5×10^5^) loaded with Calcein-AM (8μM; AnaSpec, California, USA) were added to the top chamber in media following overnight serum starvation, incubated at 37°C with 5% CO_2_ for 24h or 48h. Non-migratory cells removed from the top chamber with a cotton swab and inserts were placed in their receiver well containing trypsin with 8μM Calcein-AM and incubated for 1h at 37°C. Fluorescence was measured at 490/520 nm using the FLUOstar-OPTIMA in black well plates.

### Cell adhesion assay

Prostate cells pre-loaded with Calcein-AM were seeded at 2 × 10^4^ cells/well in black tissue culture-treated 96-well plates (Corning) pre-coated with human plasma Fibronectin (FN) (Sigma-Aldrich) or basement membrane extract (Trevigen, MD, USA) at 10μg/mL and 0.5X respectively. Plates were centrifuged at 200 rcf for 2min, incubated for 1h and read using the FLUOstar-OPTIMA plate reader (490/520 nm) to obtain an initial intensity of total cell fluorescence. Non-adherent cells were removed and fluorescence intensity of adherent cells determined.

### Statistical analysis

Statistical comparisons were performed using two-tailed, unpaired *t*-tests for CD9 mRNA, protein and cell surface levels, as well as CD9 protein levels following transfection of miRNA mimics. One-way ANOVA (Dunnett's multiple comparison testing) was used to compare *CD9* 3’UTR targeting across prostate cell lines and two-way ANOVA (Dunnett's multiple comparison testing) for analysis of cell proliferation, adhesion and migration using GraphPad Prism 6 software (San Diego, California, USA). All values are expressed as mean ± SEM and differences were considered significant at *p <* 0.05. Linear regression analysis was conducted using GraphPad Prism 6 software with a significance value of *p* < 0.05.

## SUPPLEMENTARY MATERIALS FIGURE


