# Supplementary material for: miR-518f-5p decreases tetraspanin CD9 protein levels and differentially affects non-tumourigenic prostate and prostate cancer cell migration and adhesion
Source: Oncotarget. 2017 Dec 7;9(2):1980–91. doi: 10.18632/oncotarget.23118 (PMC5788614; doi:10.18632/oncotarget.23118)
Supplement: Supplementary file 1 [file oncotarget-09-1980-s001.pdf]

miR-518f-5p decreases tetraspanin CD9 protein levels and differentially affects non-tumourigenic prostate and prostate cancer cell migration and adhesion

SUPPLEMENTARY MATERIALS

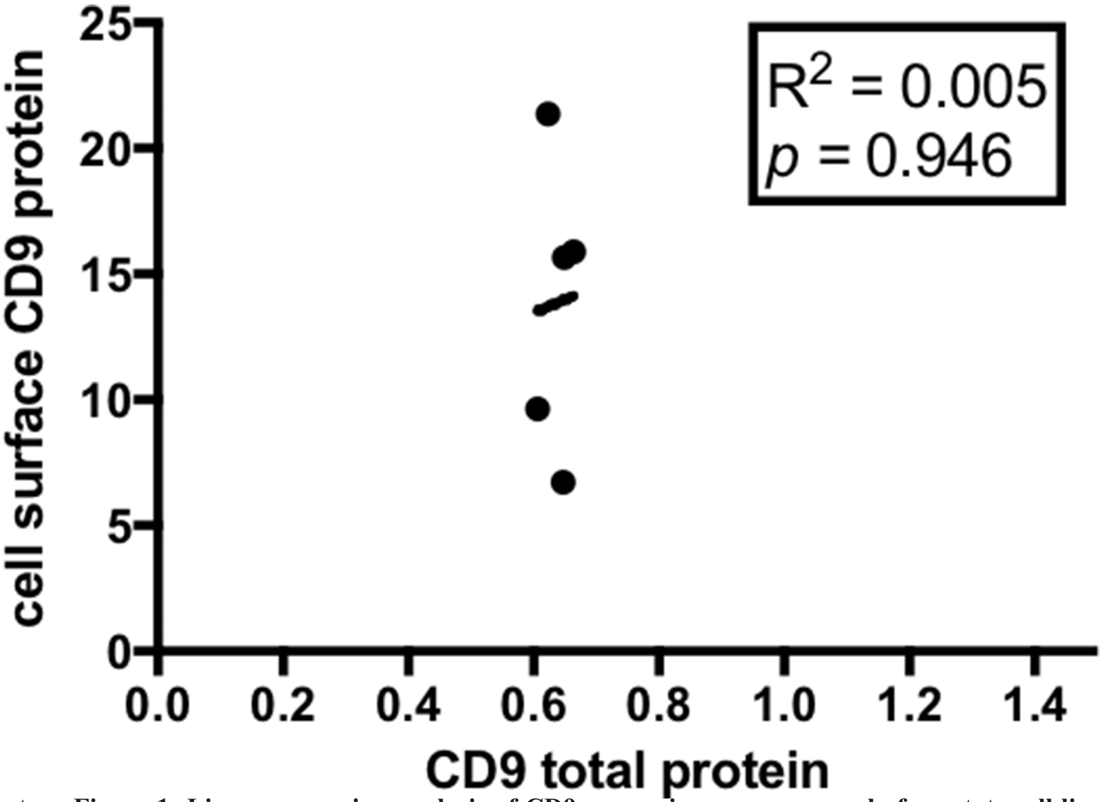

Supplementary Figure 1: Linear regression analysis of CD9 expression across a panel of prostate cell lines. Once the outlier (LNCaP) was omitted from the analysis there was no correlation between CD9 cell surface and total protein expression (vales from figure 1). Linear regression analysis was performed using Graphpad software with 95% CI and $p < 0.05$.
